# The responsibility of bioethicists: The case study of Yemen

**DOI:** 10.1111/bioe.13231

**Published:** 2023-11-02

**Authors:** Zohar Lederman, Shmuel Lederman

**Affiliations:** ^1^ Medical Ethics and Humanities Unit Hong Kong University Hong Kong; ^2^ Center of Health, Law, and Ethics Haifa University Haifa Israel; ^3^ The Weiss‐Livnat Center for Holocaust Research at Haifa University Haifa Israel

**Keywords:** cholera, human rights, Yemen

## Abstract

In this article, we describe in detail the health and general living conditions resulting from the ongoing armed conflict in Yemen, including the historical and geopolitical underpinnings. In addition to mere reporting, we use Yemen as a case study to examine the responsibility of bioethicists in general. We find it unacceptable that bioethics neglects the largest humanitarian crisis taking place in the world at the moment as well as the largest Cholera outbreak in history. We argue that bioethicists should do more to address armed conflicts and their resulting basic human rights violations. We end with a few recommendations to prevent such neglect.

“Nothing can be imagined more beautiful than the scenery of the mountains of the Yemen; Torn into all manner of fantastic peaks, the rocky crags add a wildness to a view that otherwise possesses the most peaceful charms, rich green valleys, well timbered in places, and threaded by silvery streams of dancing water; sloping fields, gay with crops and wild‐flowers; the terraced or jungle‐covered slopes,—all are so luxuriant, so verdant, that one's ideas as to the nature of Arabia are entirely upset. Well known as is, and always has been, the fertility of this region, its extent is almost startling, and it can little be wondered at that Alexander the Great intended, after his conquest of India, to take up his abode in the Yemen, had not death cut short his career”.^1^

## 1 INTRODUCTION

Armed conflicts harm people, either directly through war‐related physical and psychological injuries, or indirectly by degrading healthcare infrastructures and living conditions, displacing whole communities, and undermining people's sense of security. Healthcare professionals (HCPs) and institutions are often strategically targeted during armed conflicts, further undermining the local ability to respond to health concerns and sense of security. Malnutrition is a common theme during armed conflicts as well, and it in turn negatively affects people's health and children's cognitive and physical development.

In addition to violating the right to life and health or healthcare, armed conflicts virtually always lead to the violation of other fundamental human rights, with people being denied the option to travel freely, coerced to behave in denigrating ways, and their bodies gravely violated, particularly women's bodies. Armed conflicts endanger people's well‐being and flourishing by denying them basic human conditions such as potable water, education, electricity, a safe living environment, and so on.

Human rights violations seen from a normative rather than legal perspective are the business of social justice, and social justice that has any bearings on human health (which is plausibly always the case) is the business of bioethics. HCPs being strategically targeted during armed conflicts are also the business of bioethics.^2^ Innocent civilians being killed and injured by belligerents are the business of bioethics as well.^3^ Yet, bioethics as a field of inquiry and scholarship seems to be falling short in addressing various armed conflicts and human rights violations all over the world.

In 1967, Noam Chomsky published his classic text “The Responsibility of Intellectuals,”^4^ where he urges western intellectuals to use their privilege for the good and fight for justice while exposing governmental lies and hidden motives. Thinking principally about the American involvement in the Vietnam war, Chomsky writes: “For a privileged minority, Western democracy provides the leisure, the facilities, and the training to seek the truth lying hidden behind the veil of distortion and misrepresentation, ideology and class interest, through which the events of current history are presented to us.”

According to Chomsky, intellectuals have a responsibility to speak truth to power and to critically analyze social injustices in their historical contexts. Continuing our business without doing so is a moral failure:

“The question, “What have I done?” is one that we may well ask ourselves, as we read each day of fresh atrocities in Vietnam—as we create, or mouth, or tolerate the deceptions that will be used to justify the next defense of freedom.”

This article embodies our modest attempt to answer the question “what have I done?” We aim primarily to address a major humanitarian crisis in Yemen unfolding without due attention from (bio)ethicists. Second, we more generally aim to criticize an unfortunate gap in bioethics scholarship, namely, not paying enough attention to armed conflicts with their resultant basic human rights violations worldwide. Our strategy is to discuss the latter point first. The second part of the article will be devoted to analyzing the events unfolding in Yemen, only to end (in modesty) with concrete recommendations for the field of bioethics.

## 2 THE BUSINESS OF BIOETHICS

Our previous attempt to publish a discussion of vaccine nationalism in the context of the Israeli occupation in one of the best journals in bioethics failed as the editors (whom we highly respect) claimed that the argument made is philosophically unsophisticated, and therefore, the manuscript would be better placed in a nonacademic forum. The editors' reply merits serious discussion: should bioethics journals prioritize philosophical rigor above all, even at the cost of neglecting ongoing armed conflicts and major human rights violations? Or is there value in the mere description of such events (with a modicum of basic normative groundwork) aimed at increasing awareness in the field and paying our dues as privileged intellectuals?

A positive answer to the latter question begets another: how should concerned bioethicists choose among so many armed conflicts and grave human rights violations that are ongoing worldwide, in addition to other burning issues in bioethics? During the 16th World Congress of Bioethics and the Feminist Approaches to Bioethics Congress, the first author has noticed that at least three sessions (including one keynote) focused on “black bioethics,” calling attention to the structural injustice towards blacks in the United States. Without downplaying the importance of their message, of course, all three sessions had similar arguments. When one of the speakers was asked about what seems to be an academic redundancy (again, without doubting the importance and accuracy of their analyses and conclusions); her answer was “I write what I know.” Such reasoning is certainly reasonable as there is only so much researchers can focus on. But when an ethics conference has at least three sessions on the injustice toward one specific underprivileged population while not even mentioning the potentially largest humanitarian crisis currently unfolding in Yemen^5^; or the 1.2 million refugees, most of whom are Rohingyas, fleeing persecution in Myanmar^6^; or the 19 million Afghans suffering from extreme food insecurity^7^; or the more than 173,000 Sahrawi refugees living in five camps near Tindouf, Algeria, who are suffering from covid, extreme heat, and lack of basic needs,^8^ something has gone awry. We cannot do it all, but we should do some, and bioethicists have a responsibility to devote at least some resources to neglected armed conflicts and human rights violations worldwide. Focusing on specific cases just because they are closer to home makes sense for the researchers themselves, but not to those who suffer injustices and protest without being heard.

These two questions will be further discussed in order. But a clarificatory note is warranted first. “Bioethics” as a field of scholarship—if it is indeed a unified field of scholarship—can mean or include various different actors. Other authors, for instance, focused on funding bodies that certainly influence bioethics agenda by channeling funds where they see fit.^9^ Other actors may include clinical bioethicists who specialize in clinical ethics and are expected to engage in and write about clinical bioethics. And then there are academic bioethicists who have to maintain a research agenda while fulfilling other personal and professional duties, such as clinical consultations, practicing as clinicians, applying for grants, and making their research appealing for funding bodies. Certain subgroups within academic bioethicists may be disadvantaged compared to their peers. Female bioethicists, for instance, may indeed be under increased pressures compared to their male peers.^10^ Acknowledging all of this well, the focus here is on individual academic bioethicists as part of their professional rather than personal obligations. They may serve under various hats, including on editorial boards of journals or funding bodies, hiring committees, academic centers, and so on. They may be under immense personal and professional pressures, either from their supervisors or from funding bodies. Finally, some academic bioethicists hail from or still reside in the very places in which armed conflicts and human rights violations are occurring as we speak (write). Yet, as privileged professionals who get to write about what they care about for a living, we have a responsibility to write about what matters, and in so doing shaping our field of scholarship.^11^ While this responsibility may vary depending on the specific context in which bioethicists work, it nevertheless stands.

## 3 THE VALUE OF DESCRIPTION

Writing about armed conflicts and human rights violations means caring about human rights. By devoting the limited space that they have, bioethics journal editors—who in a sense direct the agenda for the field—make a stand. Their desire to focus on normative arguments is certainly reasonable. Normative arguments are unquestionably the bread and butter of bioethics and, together with the clarity of legal arguments, give it its analytical rigor.

Describing events and by that bringing them to wide attention has merit as well, even in bioethics. In contrast to what others have argued,^12^ normative arguments (or empirical work, for that matter) are not the only contribution that bioethics has to offer. A careful and balanced (in cases where such balance is warranted) analysis of armed conflicts may first raise awareness among bioethicists or clinicians who are too busy to read outside of their field or specific area of research. Second, the kind of clear and accessible writing expected in bioethics may facilitate the understanding of the complexity commonly inherent in armed conflicts. Third, as an academic enterprise that receives much of its support, for example, grants and salaries from public funds, bioethics has a responsibility to engage in what people care or *should* care about, and basic human rights violation certainly falls in that category. Fourth, and following the latter point, writing is caring (see below). Reporting on an event symbolizes the importance of that event, signaling to readers that they should pay attention. There is value in mere reporting, which later enables further ethical analysis and hopefully social and political activism. The fact such analysis is straightforward and does not require sophistication does not mean that it has no room in academic bioethics. On the contrary, if bioethicists wish to impact the world in significant ways, a simple argument presented succinctly in a newspaper piece or a peer‐review journal with high public visibility is much more likely to achieve such impact compared with a highly sophisticated and convoluted argument extending over a book. Lastly, and perhaps most importantly, not writing may be interpreted as not caring, and we will be judged by future generations for failing to show concern over humanitarian crises and gross human rights violations in our professional roles.

## 4 WRITING WHAT YOU KNOW

Most, if not all, bioethicists probably start their career with a genuine concern and passion toward what they write about. Most are able to maintain such concern and passion throughout their career despite increasing institutional pressures to optimize numbers, for example, impact factor and quantity of publications rather than quality. Writing about bioethics usually means writing about stuff that are or will be highly significant to people.^13^ This is a blessing and a curse of academic bioethics, or, in terms more relevant here, its privilege and responsibility.^14^

Authors are rightly concerned with academic bioethics being entangled with advocacy and activism.^15^ The former requires meticulous deliberation and an ability to engage in public reason, relying on the best available evidence. The latter is often more emotive, more populist. The two may at times be compatible, but often they are not, and when academics age in social and political activism, they may risk their professional career and respect from colleagues and readers.^16^

Our argument here, however, is much more modest. Activism and Advocacy are extremes on a spectrum, and bioethicists may and should engage the two to varying degrees either as an integral part of their scholarship or external to it.^17^ If there is something that virtually all bioethicists agree upon it is the *prima facie* value of basic human rights, however narrowly or widely they are understood. At the very least, then, bioethics should advocate the respect of basic human rights.^18^ If one cares about the basic rights of some humans qua humans, then one cares about the basic rights of all humans qua humans; anything else verges on racism or nationalism.^19^ Inequity is no longer acceptable in any shape or form, at least not by most bioethicists.^20^

To optimize effective scholarship that actually makes a change in the world, and increase the rigor of that scholarship, a detailed and comprehensive knowledge of the topic explored is essential. Bioethicists are thus very much invested in their area of research. Naturally, you invest in what you care about.^21^ Unfortunately, the nature and rapid development of the ever‐expanding topics included within bioethics mean that there is simply too much to write about: too many novel technologies, too many unreasonable policies, and too many human (or animal or environmental) rights violations and suffering.

Bioethicists should thus prioritize.^22^ The question is when such prioritization is acceptable. One can prioritize various topics, for example, end‐of‐life care or procreative freedom. In the case of armed conflicts and/or basic human rights violations, one can prioritize one event over another. One can consciously neglect some human rights violations (while hoping that others would take the lead), based on the gravity of the violation for instance, or the extent of human suffering. This kind of prioritization, however, seems to rely on value judgments that are hardly plausible—how may one human's suffering be compared to another's? One can alternatively work harder in an attempt to address all basic human rights violations. In light of their multitude in the current state of affairs, however, failure is certain. Both approaches will then inevitably lead to moral distress^23^ and frustration.

One way to bypass these challenges is to prioritize based on the amount of attention in the literature. If one cares about basic human rights, one should be invested at least to some degree in an armed conflict or an instance of human rights violation that has been neglected in the bioethics literature. Why? Because others have not and because bioethicists have a responsibility towards the victims.^24^ The passion towards and knowledge of that specific topic will soon follow, because you care about what you invest in. Put a different way, you know what you write.

In his 1997 Presidential Address, Dan Wikler describes four states that the field of bioethics has undergone. The first state consisted of professional codes of conduct; it was about medical professionals talking with and criticizing other medical professionals. The second state consisted of both medical professionals and nonprofessionals criticizing the medical institution; it was the start of the end of medical paternalism. The third state consisted of the birth of public health ethics and issues of distributive justice; this state was marked by a shift in focus from the doctor–patient relations to questions of what makes health public and what is owed to the public to facilitate health.

According to Wikler, his address at the third Congress of the International Association of Bioethics came during the birth pangs of the fourth state. This was the state of global or population health. Most relevant to us here, this is the state where bioethicists will have to prioritize on which underprivileged groups they want to focus on and which of the many injustices they want to amend:

A bioethics of population health requires, and engenders, a more vivid sense of priorities, particularly priorities for the worst off. For most people outside the Euro‐American‐Japanese world are much worse off, and those who are sick are usually at the very bottom. If we sometimes believe that we should allocate health care resources according to need, how might we also allocate bioethical energies?^25^

Wikler argues that some degree of a political or ideological agenda is compatible with rigorous and impartial scholarship, where bioethicists strive to improve the world while remaining loyal to the recognized value of rational arguments. After all, “There's little point to bioethics if we are not trying to set the world right.”^26^

Bioethicists, then, have a responsibility toward those suffering from injustices: “Bioethicists are self‐selected and have to earn the right to moralize.”^27^ Regarding what bioethicists can do exactly, Wikler writes: “We can point out issues. We can set benchmarks. And by airing and debating the issues‐‐whichever side we might advocate—we can call to account.”^28^

The downside of such an approach is that becoming an expert on a topic requires time, time that could have been invested in researching and writing about what one was inclined to write about in the first place. So, while becoming an expert on a topic that we (should) care about is indeed our responsibility as ethicists,^29^ it might not be the most efficient use of our time. For instance, the current authors are both Israelis and as such are much more comfortable writing about the Israeli occupation rather than Yemen. This downside notwithstanding, as scholars who are concerned about human lives, well‐being, and rights, we owe it to those who suffer injustices and are not being heard.

## 5 BIOETHICS AND ARMED CONFLICTS

Some of the criticism launched against bioethicists is either no longer relevant or unfair.^30^ Bioethicists are now fully cognizant of the social determinants of health and dynamics of structural violence or racism, and are extremely concerned about vulnerable populations, including people living in poverty.^31^ In other words, the “outcome gap” *is* “front and center”^32^ in bioethics. As seen during Covid, for instance, many bioethicists mobilized against vaccine nationalism based on unjust considerations or occurring in unjust circumstances.^33^

This paper argues that bioethicists should pay more attention to contemporary armed conflicts and basic human rights violations. This means engaging in political, historical, and structural analyses of such crises as others^34^ have done and as is demonstrated below. Why? Because “The primary drivers of health and its distribution are inextricably linked to historical patterns of domination, subordination, and oppression.”^35^ Our argument is not that bioethicists have completely ignored armed conflicts and grave violations of basic human rights. The first author personally knows bioethicists who worked in South Sudan, Nigeria, and other areas where conflicts were taking place. Bioethicists have addressed specific armed conflicts^36^ and the resultant migration and displacement^37^; one prominent bioethicist has even written about Yemen recently.^38^ Bioethicists have also written about various ethical dilemmas arising during armed conflicts,^39^ and may have contributed to or inspired ethical guidelines issued by humanitarian and professional healthcare organizations regarding armed conflicts in general^40^ as well as with respect to specific cases.^41^

Publications are not the only way for scholars to engage with armed conflicts and human rights violations. Bioethicists (as one reviewer rightly notes) can and indeed have addressed armed conflicts and human rights violation through conferences, workshops, and so on. An example (provided by the same reviewer) is a bid granted to Lebanon to organize a conference on bioethics and armed conflicts but which was postponed due to financial difficulties. There is nothing inherently better or worse in this kind of engagement compared to publications. What matters is the quality of scholarship and impact. Conferences might be more impactful in the local environment, engaging local stakeholders and the public, and raising awareness and interest. In the age of Zoom, the impact of conferences may be even more far‐reaching. Conversely, professional publications may be more impactful in distant locations and future times. The two should be seen as complementary.

Another way for ethicists to engage with armed conflicts and human rights violations, which could also complement professional publications and conferences, is through popular media: participating in television or radio interviews and writing in newspapers. While oftentimes the quality of scholarship is compromised in this kind of fora, their impact may be immediate and wide‐ranging.

Lastly, ethicists can also engage with these sorts of issues through social media, such as personal websites, Facebook, and so on. Especially as scholars become more prominent, this kind of engagement will become more wide‐ranging and impactful.

Our argument is simply that instances of engagement of ethicists with armed conflicts and human rights violations are few and far between. Our argument, moreover, is that ethicists tend to write about the armed conflicts and/or human rights violations that are closer to home. While this is certainly understandable, it risks neglecting groups who have no representatives in (bio)ethics. Such a neglect is wrong because no person should be neglected in her plight. Such a neglect is unjust because all members of the human community have a responsibility to care for all other members.^42^ Such a neglect is also unjust because academic (bio)ethicists as a privileged community have a responsibility towards humanity to address grave wrongs and injustices.

A discussion of the unfolding human crisis in Yemen follows as an exemplary case study. Despite being one of the greatest humanitarian crises in modern history, (bio)ethicists have devoted very little attention to it. In so doing, we failed our responsibility towards the people of Yemen. This paper is nothing but a start to amend that wrongdoing, and it aims to encourage (bio)ethicists to engage much more seriously with other humanitarian crises worldwide, particularly those cases that have been similarly neglected.

## 6 YEMEN

The Republic of Yemen, or Yemen in short, is located in Western Asia, right at the bottom of the Arab Peninsula. It borders the United Emirates to the East and Saudi Arabia to the North. Yemen's population is divided into two main religious affiliations: Zaydi Islam, a small sect of Shi'i Islam found mainly in the northern highlands, and Shafi'i Islam, a sect of Sunni Islam on the western coast and southern regions.^43^

Prior to the current armed conflict, Yemen, with its 28 million inhabitants, was already the poorest country in the Arab World. The war in Yemen is most commonly explained as a civil war between two Muslim groups divided by tribalism, religious Muslim ideologies, and political agenda: The rebel Houthis or Ansar‐Allah supported by Shiite Iran versus the Sunni government supported by the neighboring Saudi Arabia and the United Emirates, and Western countries such as the United States, Britain, and France. This explanation, however, is overly simplistic. The politics of the human tragedy in Yemen is both enlightening and confusing: one need only consider that even Israel and Al Qaeda reportedly support the Sunni government through the selling of weapons, manpower, and training.^44^ The politics is enlightening because it once again demonstrates the deleterious and destabilizing effects that global powers exert on global dynamics and people's lives.

In any event, what immediately follows is a summary of the current situation in Yemen focusing on health conditions.

Starting in September 2014, the armed conflict in Yemen has resulted in roughly 377,000 Yemeni deaths, 154,000 of whom are a direct result of the fighting while the rest resulted from hunger and preventable diseases. Of the total deaths, 259,000 were of children under the age of 5.^45^ These numbers most likely represent an underestimation, as many Yemenis are dying in their houses, especially in remote villages, without anyone counting them; most recently, the number of deaths has been estimated to be over a million.^46^

Nearly 24 million Yemenis are in need of humanitarian assistance, including 12.9 million children.^47^ Nineteen million people are specifically suffering from food insecurity.^48^ More than 2 million children under the age of five and about 1.3 million pregnant and lactating women are projected to suffer from acute malnutrition in 2022.^49^

According to the United Nations Office for the Coordination of Humanitarian Affairs (UNOCHA),

Though Yemen had pre‐existing vulnerabilities, the conflict's increasingly protracted nature has resulted in economic collapse, increased poverty and the breakdown of national social‐protection systems and community safety nets. It has exacerbated long‐standing vulnerabilities and severely frayed Yemen's social fabric. Loss of revenues, depreciation of the Yemeni rial and import restrictions resulted in loss of income and rising prices for most basic household items, including food. Millions of people are now in a situation where they can no longer meet their basic needs, with potential serious detrimental impacts on groups in society with limited social capital and protection mechanisms. This increases risks of adopting harmful coping strategies, such as debt, selling of assets, early/forced marriages, school dropout and child labor, with grave long‐term impacts especially on women, children, older persons, persons with disabilities and marginalized communities.^50^

## 7 HEALTH CONDITIONS IN YEMEN

According to a September 2022 UNOCHA report, Yemen “is already one of the world's largest humanitarian crises.”^51^ Death and disease are commonplace. Eleven children were killed in May 2022 alone, either intentionally by various parties to the conflict or due to gunshot accidents.^52^

Basic human rights in Yemen are continuously being violated, mostly but not solely by the Saudi‐led coalition. People in Yemen, particularly women, are concerned for their safety and the safety of their families. Violence against women seems to be increasing, and girls are being married at a young age for financial purposes: two‐thirds of Yemeni girls under the age of 18 are married.^53^ Ten million children and 5 million women lack proper access to healthcare,^54^ and 4.3 million people are internally displaced.^55^ The lack of access to healthcare stems partially from the lack of healthcare facilities, as no less than 133 attacks on medical facilities have been documented in Yemen since April 2014.^56^ Most strikingly, some hospitals were attacked several times, making such attacks unlikely to be accidental.^57^ Another reason for the lack of access is the absence of prehospital Emergency Medical Services.^58^

Yemen is also suffering from various infectious disease outbreaks, including measles, cholera, Diphtheria, Dengue, and of course Covid‐19.^59^

Early warnings of a measles epidemic due to the conflict and reduced vaccine coverage to mere 41% of the population^60^ turned out to be accurate, and up until May 2022, 2924 cases were confirmed, with 34 deaths.^61^

Yemen is also experiencing the most severe cholera epidemic in history, with over 1 million cases and 3000 deaths.^62^ According to UNICEF's latest report, 8500 people suffered from Acute Watery Diarrhoea (AWD)/suspected cholera epidemic in the first half of 2022 alone. Cholera is transmitted via the fecal–oral route, and such a high prevalence indicates lack of clean and safe potable water; more than 70% of people in Yemen are estimated to lack access to safe drinking water.^63^ A safe and relatively effective oral vaccine for cholera exists, but lack of healthcare infrastructure and available vaccines in Yemen hampered its delivery. Interestingly, the slow mobilization of the international humanitarian community was criticized by biomedical scientists^64^ but not even mentioned by bioethicists. Aligned with the spirit of the current paper, these scientists in fact urge academics to act:

…public health professionals and academics are in a strong position to join in by applying their knowledge and skills in advocacy efforts for peace and the preservation of public health in conflict and to keep the world's leaders accountable for the health and human rights consequences of their actions.^65^

Up until the second half of 2022, roughly 12,000 people were confirmed to have Covid‐19, and 18% of them succumbed.^66^ As of writing, only 2.9% of the population received at least one dose of the vaccine.^67^

While being endemic to Yemen, an epidemic of Diphtheria was announced in Yemen in 2017. Up to April 2020, 5701 probable cases and 330 deaths were recorded, making it the largest outbreak of diphtheria in the past 20 years.^68^ The vaccine rate in Yemen remains less than 80%, which is the necessary threshold for herd immunity. A children vaccination campaign began in November 2018, but was hampered due to the conflict. The outbreak could have been prevented with existing vaccines known to be safe and effective and deaths could have been prevented using the diphtheria antitoxin, but neither is currently readily available in Yemen. Similar to Covid and Cholera, then, morbidity and mortality could have been prevented with resources that are readily available in the Western world, but not in Yemen.

In addition to measles, Diphtheria, Covid, and Cholera, Yemen is also experiencing an outbreak of circulating Vaccine Derived Polio Virus (cVDPV), with 95 confirmed cases so far.^69^

Lastly, in the background, the effects of climate change pose a significant threat to the life, well‐being, and prosperity of people in Yemen.^70^ During 2022 alone, Yemen suffered from a severe drought during the first half of the year, followed by extreme flooding. These extreme weather conditions led to the loss of crops, thus increasing food insecurity, destruction of housing, and migration of undetonated artillery closer to residential areas, decreasing housing security. The flooding also damaged roads, property, and farms, affecting peoples' livelihoods and ability to travel.

According to the UNOCHA, US4.3$ billion are needed to attend to the needs of people in Yemen; as of September 2022, it has received merely 2 billion.^71^

A United Nations‐brokered 6‐month truce between the Houthis and Saudi‐led coalition ended on October 2, 2022. During the ceasefire, civilian deaths declined by 60% and displacement declined by 50%.^72^ Unlike his predecessors Barak Obama and Donald Trump, the Biden administration has been less active in collaborating with the Saudis and more active in ending the conflict.^73^ In his recent visit to Saudi Arabia, Biden could have urged Muhammed Bin Salman to end the war but did not.^74^ Among other reasons, he did not because there is not enough public pressure to do so, and this is partially on bioethicists who only write what they know, choosing to neglect the largest cholera outbreak in history as well as the largest ongoing humanitarian crisis in the world.

The ongoing onslaught in Yemen must end. It is an unjust war and innocent citizens, including children, are being harmed. HCPs and institutions are being targeted. Both sides are culpable, as are all the countries and organizations that support them militarily.^75^

## 8 CONCLUDING REMARKS

Armed conflicts find their origins in the past, harm people in the present, and threaten generations well into the future. They virtually always entail basic human rights violations and injustices. Bioethicists should strive to become experts on one or more of the many ongoing conflicts and keep them high on the bioethics agenda. Bioethics as a field of inquiry must remain relevant and must advocate basic human rights, human health, and human well‐being. Remaining relevant means not neglecting what is potentially the largest humanitarian crisis that humankind is currently experiencing.

Sadly, in the end, we must conclude that the imprint of empire on Yemen takes its most enduring form in graves, bombed medieval cities, and a whimper from starving children no one want to hear. And with this stark reality, I have nothing else to do. This is the end is just a book.^76^

And this is just a paper. We have argued that academic bioethics ought to pay more attention to neglected armed conflicts and basic human rights violations. We have further argued that bioethicists have a responsibility to engage events occurring in contexts that they do know. We have focused on the humanitarian crisis unfolding in Yemen to get our point across.

When all is said and done, reality cannot be ignored. Both junior and senior Bioethicists face significant pressure from deans, center directors, search committees, or grant sponsors to focus their research and optimize its impact. They may thus be discouraged from writing about armed conflicts that have never been or no longer are on primetime media such as Yemen or the Israeli occupation. In light of the discussion so far, however, bioethicists should nevertheless persist.

Junior bioethicists should take some professional risks in attending to armed conflicts, whether it means devoting precious resources or engaging in controversial topics. More senior bioethicists including journal editors should rather encourage such advocacy, perhaps by way of the following tentative recommendations:

1. Academic search committees as well as grant sponsors should emphasize the importance of the kind of advocacy mentioned here.
2. Graduate programs should encourage and devote time to this kind of advocacy (as some of them already do, as pointed out by a reviewer). One specific strategy may be collaboration with local or international humanitarian organizations as part of the training.
3. Journal editors should in general encourage more advocacy of this kind, for example, by enabling special issues on armed conflicts (like the current one) or soliciting individual projects by bioethicists.
4. Bioethics organizations such as the International Association of Bioethics should encourage this kind of advocacy, for instance, by soliciting workshops or conference panels (as the mentioned Lebanon bid) during its international Congress.
5. As could already be seen to some degree, academic scholars should closely engage policy‐makers, through joint workshops, publication of white papers, and so on.

## 9 POSTSCRIPT

More than 6 months have elapsed since this paper was first drafted, and in one sense, the situation in Yemen seems to be improving. A China‐brokered negotiation between Saudi Arabia and Iran is underway, and civilian life is beginning to normalize. Commercial flights and sea commerce have been reinstated, and instances of military hostility have been reduced, albeit not disappeared completely.^77^

Health conditions, however, are still dire. According to a WHO assessment in April 2023, up to 31.5 million people are in acute need of humanitarian and protection services. “540,000 Yemeni children under age five are currently suffering from severe acute malnutrition with a direct risk of death. 46 percent of health facilities across the country are only partially functioning or completely out of service due to shortages of staff, funds, electricity, or medicines.” Funding coming from the international community to address such acute needs in Yemen has been utterly insufficient. The impact of preventable infectious diseases is worsening: “In the first quarter 2023, more than 13,000 new cases of measles, 8,777 cases of dengue fever, and 2,080 suspected cholera cases were reported. But the actual numbers are likely much higher, due to gaps in surveillance system.” This time of year is marked by flooding, which is bound to increase the prevalence of vector‐borne diseases even further.^78^

A report issued in May 2023 by the United Nations Office for the Coordination of Humanitarian Affairs summarizes the events unfolding in Yemen in 2022. Social restrictions in Houthis‐controlled areas have worsened, including Islamification of education curriculum, stricter dress codes, prohibition of reproductive health services, and increased segregation between men and women in universities and cafes.^79^ These all mean, in essence, increased social control of men over women and children.

The sense of security in Yemen has also been diminishing. More deaths in 2022 were attributed to explosive ordnance rather than gunshot wounds, which means that people living in rural areas with higher prevalence of such ordnance feel less safe for their lives and their agricultural property. The need to take transportation routes that are perceived to be safer often entails greater costs. People injured by landmines may become dependent on family members, resulting in social isolation for the individual as well as the household. The humanitarian crisis in Yemen, in other words, is far from being resolved, and fellow humans are unjustly suffering.

All we write about in bioethics is urgent and important, but some topics are more urgent and important than others.

## Footnotes

1 Harris, W. B. (1893). *A journey through the Yemen and some general remarks Upon that country* (pp. 102–106). William Blackwood and Sons. Kindle Locations.

2 Lederman, Z. (2021). Together we lived, and alone you died: Loneliness and solidarity in Gaza. *Developing World Bioethics*, *21*, 17–24; Rubenstein, L., & Haar, R. (2022). What does ethics demand of health care practice in conflict zones? *AMA Journal of Ethics*, *24*, E535–E541.

3 WMA resolution in support of medical personnel and citizens of Ukraine in the face of The Russian Invasion. 2022, WMA Resolution in Support of Medical Personnel and Citizens of Myanmar. 2021.

4 Chomsky, N. (1967). *The responsibility of intellectuals*. The New York Review of Books.

5 United Nations Population Fund. (2023). *Yemen: The world's largest humanitarian crisis* (unfpa.org). Retrieved October 2022, from https://www.unfpa.org/yemen-crisis-women-and-girls

6 UNCHR. *Myanmar situation* (unhcr.org). Retrieved October 2022, from https://reporting.unhcr.org/operational/situations/myanmar-situation#:~:text=2022%20Situation%20overview,urgent%20need%20of%20humanitarian%20assistance

7 News. *As the world watches Russia's war, other global crises see critical support fading* (nbcnews.com) Retrieved October 2022, from https://www.nbcnews.com/news/world/russia-war-ukraine-drains-aid-crisis-afghanistan-yemen-horn-africa-rcna29169

8 ReliefWeb. ACAPS Briefing Note: Algeria: Sahrawi refugees in Tindouf (January 19, 2022)—Algeria. Retrieved October 2022, from https://reliefweb.int/report/algeria/acaps-briefing-note-algeria-sahrawi-refugees-tindouf-19-january-2022

9 Fabi, R., & Goldberg, D. S. (2022). Bioethics, (Funding) Priorities, and the Perpetuation of Injustice. *The American Journal of Bioethics, 22*, 6–13. Goering, S., et al. (2022). Integrating equity work throughout bioethics. *The American Journal of Bioethics, 22*, 26–27.

10 Fabi & Goldberg, op. cit. note 9.

11 Wilson, Y. Y. There are priorities and then there are priorities: A prior question about the perpetuation of injustice through bioethics research funding. *American Journal of Bioethics*, *22*(1), 19–21; Ibid: 19–21.

12 Millum, J. (2022). How to identify priority questions for bioethics research. *The American Journal of Bioethics, 22*, 17–18.

13 Scully, J. L. (2019). The responsibilities of the engaged bioethicist: Scholar, advocate, activist. *Bioethics*, *33*, 872–880.

14 Ibid; Fins, J.J. (2022). Bioethics, Ukraine, and the Peril of Silence. *Cambridge Quarterly of Healthcare Ethics, 32*, 1–3.

15 Benatar, D. (2006). Bioethics and health and human rights: A critical view. *Journal of Medical Ethics, 32*, 17–20; Cribb, A. (2010). Translational ethics? The theory–practice gap in medical ethics. *Journal of Medical Ethics, 36*, 207.

16 See, for instance, the case of Ken Roth. Free speech seems to see different standards for anti‐Israel criticism | The Mehdi Hasan Show—YouTube. Retrieved February 2023, from https://www.youtube.com/watch?v=02waZ33ZJQs&t=1s, or the most recent case of legal scholar James Cavallaro. Silencing critics of Israel: Biden pulls nomination of human rights lawyer for decrying apartheid. Democracy Now! Retrieved February 2023, from https://www.democracynow.org/2023/2/17/james_cavallaro_state_department_israel_criticism

17 Rogers, W. (2019). Bioethics and activism: A natural fit? Bioethics, *33*, 881–889; Scully, op. cit. note 13.

18 Fabi & Goldberg, op. cit. note 9.

19 Venkatapuram, S. (2021). Global health without justice or ethics. *Journal of Public Health, 43*, 178–179; Benatar, S., & Brock G. (2011). Global health ethics: The rationale for mutual caring. In G. Brock & S. Benatar (eds), *Global health and global health ethics* (pp. 129‐140). Cambridge University Press; Fins, op. cit. note 14.

20 For a critique against some who do seem to treat a certain population in Israel inequitably, see Lederman, Z., & Lederman, S. (2017). The land of no milk and no honey: Force feeding in Israel. *Monash Bioethics Review, 34*, 158–188.

21 Frankfurt, H. (1982). The importance of what we care about. *Synthese, 53*, 257–272.

22 Fabi & Goldberg, op. cit. note 9.

23 Campbell, S. M., Ulrich, C. M., & Grady, C. (2016). A broader understanding of moral distress. *The American Journal of Bioethics, 16*, 2–9.

24 Rogers, W. A., & Scully, J. L. (2021). Activism and bioethics: Taking a stand on things that matter. *Hastings Center Report, 51*, 32–33; Scully, op. cit. note 13.

25 Wikler, D. (1997). Presidential address: Bioethics and social responsibility. *Bioethics, 11*, 185–192. p. 187.

26 Ibid: 188.

27 Ibid: 192.

28 Ibid.

29 Pogge, T. W. (2008). *World poverty and human rights* (2nd ed.). Polity.

30 Farmer, P., & Campos, N.G. (2004). Rethinking medical ethics: A view from below. *Developing World Bioethics, 4*, 17–41.

31 Rogers, op. cit. note 17.

32 Farmer, op. cit. note 30.

33 Jecker, N. S., & Lederman, Z. (2022). Three for me and none for you? An ethical argument for delaying COVID‐19 boosters. *Journal of Medical Ethics*, *48*, 662–665; medethics‐2021‐107824; Schaefer, G.O., Leland, R. J., & Emanuel, E. J. (2021). Making vaccines available to other countries before offering domestic booster vaccinations. *JAMA*, *326*(10), 903–904; Lederman, Z., Lederman, S., & Majadli, G. (2022). Covid‐19 in the occupied palestinian territory and Israel's duty to vaccinate. *Journal of Jewish Ethics*, *8*(2), 177–206. https://doi.org/10.5325/jjewiethi.8.2.0177

34 Farmer, P. (2003). *Pathologies of power: Health, human rights, and the new war on the poor*. University of California Press; Farmer, P. (2020). *Fevers, feuds, and diamonds: Ebola and the ravages of history*. Farrar, Straus and Giroux.

35 Fabi & Goldberg, op. cit. note 9.

36 Fins, op. cit. note 14.

37 Klingler, C., Odukoya, D., & Kuehlmeyer, K. (2018). Migration, health, and ethics. *Bioethics*, *32*, 330–333.

38 Rubenstein, L. (2021). *Perilous medicine*. Columbia University Press.

39 Arawi, T., & Abu‐Sittah, G. S. (2022). Bioethics in conflict zones. *AMA Journal of Ethics, 24*(6), E455–E456; O'Mathúna, D., & Upadhaya, N. (2022). Should children be enrolled in clinical research in conflict zones? *AMA Journal of Ethics*, *24*, E463–E471; Arawi, T., Abu‐Sittah, G. S., Hassan, B. (2022). Everyone is harmed when clinicians aren't prepared. *AMA Journal of Ethics*, *24*, E489–E494; Reynolds C. W., & Meertens. C. S. (2022) How Should Health Systems Help Clinicians Manage Bias Against Ex combatants? *AMA Journal of Ethics*, *24*, E483‐E488; Rubenstein & Haar, op. cit. note 2.

40 The International Committee of The Red Cross. (2015). Ethical principles of health care in times of armed conflict and other emergencies. The International Committee of The Red Cross.

41 WMA resolution in support of medical personnel and citizens of Myanmar, wma resolution in support of medical personnel and citizens of Ukraine in the face of the Russian invasion.

42 Stauffer, J. (2015). *Ethical loneliness: The injustice of not being heard*. Columbia University Press; Have, H.T. (2016). *Vulnerability*. Routledge.

43 Orkaby, A. (2021). *Yemen what everyone needs to know*. Oxford University Press; Boucek, C. (2010). War in saada from local insurrection to national Challenge; Riedel, B. (2017). Who are the Houthis, and why are we at war with them? *Brookings*. The original submission included much more extensive historical background, but this was discarded at the request of the reviewers. Interested readers may ask us for that background or consult the relevant literature: Yemen | Situation Reports (unocha.org). Retrieved October 2022, from https://reports.unocha.org/en/country/yemen. Yemen's war is a mercenary heaven. Are Israelis reaping the profits?—Haaretz Com-Haaretz.com. Retrieved October 2022, from https://www.haaretz.com/2019-02-17/ty-article/.premium/yemens-war-is-a-mercenary-heaven-are-israelis-reaping-the-profits/0000017f-e139-d75c-a7ff-fdbdee4b0000. Yemen | History, map, flag, population, capital, & facts | *Britannica*. Retrieved October 2022, from https://www.britannica.com/place/Yemen. Harris. Orkaby, I. Blumi. (2018). *Destroying Yemen: What chaos in Arabia tells us about the world*. California Press. Burrowes, R. D. (2005). The famous forty and their companions: North Yemen's first‐generation modernists and educational emigrants. *Middle East Journal*, *59*, 81–97.

44 Haaretz Com, op. cit. note 4.

45 Hanna, T., et al. (2021). The United Nations Development Programme (UNDP). Assessing the impact of war in Yemen: Pathways for recovery.

46 6/3/22 Aisha Jumaan on How You can Help End the War Against Yemen. Retrieved October 2022, from https://scotthorton.org/interviews/6-3-22-aisha-jumaan-on-how-you-can-help-end-the-war-against-yemen/

47 Health | UNICEF Yemen. Retrieved October 2022, from https://www.unicef.org/yemen/health; Yemen | Situation Reports, op. cit. note 43.

48 Yemen | Situation Reports, op. cit. note 43.

49 Yemen Humanitarian Situation Report, 31 May 2022 | UNICEF. Retrieved October 2022, from https://www.unicef.org/documents/yemen-humanitarian-situation-report-31-may-2022; Biden decision to stop support of Saudi‐led war in Yemen greeted with cautious relief (nbcnews.com). Retrieved October 2022, from https://www.nbcnews.com/news/world/biden-decision-stop-support-saudi-led-war-yemen-greeted-cautious-n1256868

50 Yemen | Global Humanitarian Overview (unocha.org). Retrieved October 2022, from https://humanitarianaction.info/

51 Yemen | Situation Reports, op. cit. note 43.

52 Unicef. (2022). *Yemen Country Office Humanitarian Situation Report*. Unicef.

53 The Women Fighting to Protect Yemen—YouTube. Retrieved October 2022, from https://www.youtube.com/watch?v=dbU37lo5fV4&t=4s

54 Unicef. Yemen Country Office Humanitarian Situation Report.

55 Yemen | Situation Reports, op. cit. note 43.

56 Medical Facilities Under Fire in Yemen (yemeniarchive.org). Retrieved October 2022, from https://medical-facilities.yemeniarchive.org/

57 Ibid.

58 Alsabri, M., Alhadheri, A., Alsakkaf, L. M., & Cole, J. (2021). Conflict and COVID‐19 in Yemen: beyond the humanitarian crisis. *Globalization and Health*, *17*, 83.

59 Humanitarian crisis in Yemen: Seven years on, WHO is strengthening the health system, responding to vital health needs, and saving lives—Yemen | ReliefWeb. Retrieved October 2022, from https://reliefweb.int/report/yemen/humanitarian-crisis-yemen-seven-years-who-strengthening-health-system-responding-vital; ibid.

60 Qirbi, N., & Ismail, S.A. (2016). Ongoing threat of a large‐scale measles outbreak in Yemen. *The Lancet Global Health*, *4*, e451.

61 2900 cases of measles in Yemen—Al‐Mashhad Net (almushahid.net). Retrieved October 2022, http://almushahid.net/95964/. UNICEF and WHO warn of ‘perfect storm’ of conditions for measles outbreaks, affecting children Retrieved October 2022, from https://reliefweb.int/report/yemen/humanitarian-crisis-yemen-seven-years-who-strengthening-health-system-responding-vital

62 Ng, Q. X., De Deyn, M. L. Z. Q., Loke, W., & Song Yeo, W. (2020). Yemen's cholera epidemic is a one health issue. *Journal of Preventive Medicine and Public Health, 53*, 289–292; Federspiel, F., & Ali, M. (2018). The cholera outbreak in Yemen: Lessons learned and way forward. *BMC Public Health, 18*, 1338; Cholera in Yemen: What to Know About the Outbreak | Time. Retrieved October 2022, from https://time.com/4874345/yemen-cholera-outbreak/

63 Alsabri, M., et al., op. cit. note 58.

64 Federspiel & Ali, op. cit. note 62; Zaidi, A. (2017). Make plans to eliminate cholera outbreaks. *Nature*, *550*, 9.

65 Federspiel & Ali, op. cit. note 62.

66 Unicef. Yemen Country Office Humanitarian Situation Report. 18% mortality seems exaggerated for Covid. One would assume that the overall prevalence was much higher but just not confirmed.

67 Understanding Vaccination Progress by Country—Johns Hopkins Coronavirus Resource Center (jhu.edu). Retrieved October 2022, from https://coronavirus.jhu.edu/vaccines/international

68 Badell, E., et al. (2021). Ongoing diphtheria outbreak in Yemen: A cross‐sectional and genomic epidemiology study. *The Lancet Microbe*, *2*, e386–e396.

69 Yemen Humanitarian Situation Report, op. cit. note 49.

70 In this article, we focus on human beings. Armed conflict and climate change affect animals and ecosystems as well, however, and they should not be neglected. The severe drought in the country, for instance, significantly limited the food available to livestock. Yemen | Situation Reports, op. cit. note 43.

71 Yemen | Situation Reports, op. cit. note 43.

72 End of Yemen's truce leaves civilians afraid dark days are back | News | Al Jazeera. Retrieved October 2022, from https://www.aljazeera.com/news/2022/10/7/end-yemen-truce-leaves-civilians-afraid-dark-days-back

73 Biden decision to stop support of Saudi‐led war in Yemen greeted with cautious relief (nbcnews.com) Retrieved October 2022, from https://www.nbcnews.com/news/world/biden-decision-stop-support-saudi-led-war-yemen-greeted-cautious-n1256868

74 Biden Can Use His Saudi Trip To Help End the Yemen War | Time. Retrieved October 2022, from https://time.com/6195677/biden-saudi-trip-yemen/

75 Rubenstein, op. cit. note 28.

76 Blumi, op. cit. note 43, p. 211.

77 UN envoy reports on progress towards Yemen peace agreement | UN News. Retrieved May 2023, from https://news.un.org/en/story/2023/05/1136752

78 WHO update on health situation and needs in Yemen at today's UN bi‐weekly press briefing in Geneva—Yemen | ReliefWeb. Retrieved May 2023, from https://reliefweb.int/report/yemen/who-update-health-situation-and-needs-yemen-todays-un-bi-weekly-press-briefing-geneva

79 ACAPS Thematic report: Yemen—Social Impact Overview 2022 (05 May 2023)—Yemen | ReliefWeb. Retrieved May 2023, from https://reliefweb.int/report/yemen/acaps-thematic-report-yemen-social-impact-overview-2022-05-may-2023

